# Efficient Incorporation of DOPA into Proteins Free from Competition with Endogenous Translation Termination Machinery

**DOI:** 10.3390/biom15030382

**Published:** 2025-03-06

**Authors:** Youhui Yang, Yingchen Wang, Zhaoguan Wang, Hao Qi

**Affiliations:** 1 School of Chemical Engineering and Technology, Tianjin University, Tianjin 300072, China; yangyouhui@tju.edu.cn (Y.Y.); yingchen196@126.com (Y.W.); wzg1895@tju.edu.cn (Z.W.); 2 Key Laboratory of Systems Bioengineering (Ministry of Education), Tianjin University, Tianjin 300072, China

**Keywords:** noncanonical amino acids, 3,4-dihydroxy-L-phenylalanine, sfGFP, aminoacyl-tRNA synthetase

## Abstract

3,4-Dihydroxy-L-phenylalanine (DOPA) is a promising noncanonical amino acid (ncAA) that introduces novel catechol chemical features into proteins, expanding their functional potential. However, the most common approach to incorporating ncAAs into proteins relies on stop codon suppression, which is often limited by the competition of endogenous translational termination machinery. Here, we employed a special in vitro protein expression system that facilitates the efficiency of DOPA incorporation into proteins by removing essential Class I peptide release factors through targeted degradation. In the absence of both RF1 and RF2, we successfully demonstrated DOPA incorporation at all three stop codons (TAG, TAA, and TGA). By optimizing the concentration of engineered DOPA-specific aminoacyl-tRNA synthetase (DOPARS), DOPA, and DNA template, we achieved a synthesis yield of 2.24 µg of sfGFP with 100% DOPA incorporation in a 20 μL reaction system. DOPARS exhibited a dissociation constant (*Kd*) of 11.7 μM for DOPA but showed no detectable binding to its native counterpart, tyrosine. Additionally, DOPA was successfully incorporated into a reverse transcriptase, which interfered with its activity. This system demonstrates a fast and efficient approach for precise DOPA incorporation into proteins, paving the way for advanced protein engineering applications.

## 1. Introduction

3,4-Dihydroxy-L-phenylalanine (DOPA), a noncanonical amino acid (ncAA) derived from the post-translational modification of tyrosine, is widely distributed in nature and has diverse applications. Inspired by the strong adhesion properties of mussel foot proteins, DOPA-based materials have been developed for chiral bionic pigments with robust adhesion and high stability [1], a novel facile antifouling coating [2], and antipathogenic biomaterials [3]. Besides its applications in biomaterials, DOPA serves as a sensor for metal ion detection through ion coordination [4,5] and functions as a catalytic radical in certain enzymes [6]. To incorporate DOPA into proteins, several strategies have been developed for producing recombinant proteins containing DOPA, including the global suppression of tyrosine residues using the tyrosine auxotrophic hosts (*E. coli* JW2581 strain) [5] and enzymatic modification of exposed tyrosine residues [2].

Genetic code extension (GCE) enables site-specific incorporation of ncAAs into proteins via orthogonal aaRS/tRNA pairs that reassign nonsense or frameshift codons, bypassing endogenous translation mechanisms [7]. While stop codon suppression is widely used for ncAA incorporation, efficient DOPA incorporation remains challenging due to competition from endogenous release factors (e.g., RF1 and RF2) and limitations in cellular uptake. Several site-specific methods of incorporating DOPA into proteins using GCE have been reported. To enhance the fidelity of DOPA incorporation, a photocaged analogue was synthesized and employed within GCE to incorporate the photocaged analogue into the target protein. Subsequently, UV irradiation was utilized to liberate the DOPA-containing protein [8,9]. However, this approach requires the synthesis of a photocaged noncanonical amino acid, which incurs higher costs. To address the persistent competition from endogenous release factors, the researchers used codon redistribution to replace all amber codons in C321.ΔA [10] and B95.ΔA [11], and peptide chain release factor RF2 (*prfB*) was removed from the genome, increasing the incorporation efficiency of ncAAs. Meanwhile, to facilitate the incorporation of ncAAs, the syn61 strain was achieved by compressing a redundant codon functionality, liberating three essential codons for reassignment [12]. However, the stability of the genome and the growth rate of the strain still face some challenges. The intracellular synthesis of proteins containing ncAAs faces challenges such as the uptake of ncAAs and difficulties regulating protein expression, limiting its widespread application. To address these issues, the cell-free unnatural protein synthesis (CFUPS) system provides a viable alternative. Advancements in CFUPS, particularly in flexibility and openness, have facilitated the development of CFUPS systems [13]. Recently, the Lon protease from the *Mesoplasma florum* (*mf*-Lon) has been demonstrated to specifically target proteins for efficient degradation in *Escherichia coli* [14]. Thus, CFUPS was combined with *mf*-Lon’s degradation properties to develop a Class I release factor-free system for efficient ncAA incorporation [15].

In this study, we employed a novel system, the Class I release factor-free CFUPS, by leveraging the specific degradation effect of *mf*-Lon on protein degradation tag 3 (pdt3)-tagged proteins. By meticulously optimizing the concentrations of DOPARS, template, and ncAAs in the system, we improved the expression of the DOPA-containing protein. Furthermore, the fidelity of DOPA incorporation was rigorously validated through mass spectrometry, and the underlying mechanism was preliminarily explored through isothermal titration calorimetry (ITC) analysis. Finally, we successfully incorporated DOPA into the FrM4 protein. This study introduces a novel approach to enhance the expression of proteins comprising unnatural amino acids, which is anticipated to drive significant progress in biomedical research and drug development.

## 2. Materials and Methods

### 2.1. Plasmid Construction

The DOPARS (L65E, H70A, Y119F, D158A, I159A, E220K, F261S, N268D, L282P, H283P, P284L, M285R and D286A) [16] (GenBank: QLK97558.1) from *Methanocaldococcus jannaschii* and the Friend murine leukemia virus reverse transcriptase (FrM4) (GenBank: Z11128.1) containing four mutations (D178C, E280R, T284R, and L581W) (unpublished data) were codon-optimized for *E. coli* and synthesized by BGI Tech Solutions Co., Ltd. (Beijing, China, Liuhe). Subsequently, the gene was amplified with PrimeSTAR Max DNA Polymerase using specific primers (Supplementary Table S1) and ligated into the linearized pET28a and pUC19 backbone using a ClonExpress Ultra One Step Cloning Kit V2 (Vazyme #C116-01). The pET23a-sfGFP, pET23a-sfGFP149TAG, and pZA16mflon-*Mj*tRNA^DOPA^_CUA_ plasmids were stored in our lab. All the plasmid constructs were verified by Sanger sequencing (Suzhou, China, Azenta).

### 2.2. Genome Editing

The BL21(DE3) strain was subjected to genome editing using λ-Red-mediated homologous recombination following the procedures detailed in our previous study [15]. Briefly, a pdt3 tag [14] was inserted into the 3′ end of the *prfA* and peptide chain release factor RF2 (*prfB*) gene. To enhance the detection of protein expression and degradation, a flag tag was added before the pdt3 tag. A flag tag was also incorporated at the 3′ end of the glyceraldehyde-3-phosphate dehydrogenase (*gapA*) gene. To mitigate potential interference from the endogenous Lon protein, the small stable RNA A (*ssrA*) gene has been deleted from the BL21(DE3) strain. The sequences of all edited genomic regions were validated through Sanger sequencing. Successful genome editing yielded the BL21(DE3) S-AAF3BF3 strain.

### 2.3. Extract Preparation

The BL21(DE3) S-AAF3BF3 strain was transformed with the suppression plasmid (pZA16mflon-*Mj*tRNA^DOPA^_CUA_) and cultured overnight at 37 °C in 2× YTP medium (16 g/L of tryptone, 10 g/L of yeast extract, 5 g/L NaCl, 20 mM KH_2_PO_4_, 40 mM K_2_HPO_4_). The culture was then diluted 1:100 into fresh 2× YTP medium and grown to an OD600 of 0.6 at 30 °C with 1 mM IPTG. The cells were induced with 1 mM L-arabinose at 37 °C for 8 h. The cells were harvested by centrifugation at 4,200 rpm for 30 min. The pellet was washed two times with cold S30 buffer (30 mM potassium glutamate, 10 mM Tris-acetate pH 8.2, 2 mM DTT, 14 mM magnesium acetate), and the wet weight was measured. The pellet was resuspended in 1 mL S30 buffer per 1.1 g wet weight of cells before lysis. The suspension was lysed using a high-pressure cell cracker (JNBIO #JN-Mini Pro, Shenzhen, China) at 1,000 bar in an ice bath. To remove the pellet, the lysate was centrifuged twice at 30,000× *g* for 30 min and 4 °C. The supernatant was then subjected to a run-off reaction (37 °C, 220 rpm, 30 min) and centrifuged as described previously. The supernatant was collected, frozen in liquid nitrogen, and stored at −80 °C.

### 2.4. Western Blot

The protein was collected and mixed with 5× sample loading buffer (300 mM Tris-HCl pH 6.8, 12.5% (*w*/*v*) SDS, 10‰ (*w*/*v*) bromophenol blue, 50% (*v*/*v*) glycerol, 0.5 M DTT) and then denatured at 98 °C for 10 min. The samples were separated by 10% SDS-PAGE and transferred to PVDF membranes (Merck #IPVH08100, Boston, MA, USA). The membranes were blocked with 5% skim milk overnight at 4 °C. Subsequently, the membranes were incubated with primary antibodies (anti-flag (#AE005), anti-Twin strep (#AE066); ABclonal, Wuhan, China) at 25 °C for 1 h. Subsequently, the membranes were incubated with secondary antibody (Thermo Fisher #31430, Waltham, MA, USA) at 25 °C for 1 h. Finally, the membrane was treated with SuperSignal™ West Pico PLUS Chemiluminescent Substrate (Thermo Fisher #34580, MA, USA) for imaging.

### 2.5. Expression and Purification of the Proteins

The DOPARS with a C-terminal His-tag was expressed in *Escherichia coli* BL21(DE3) [*F−ompT hsdSB* (*rB−*, *mB−*) *galdcmrne131*(DE3)] cells. A single colony was placed in an LB medium containing 50 µg/mL of kanamycin and incubated overnight. Then, the culture was diluted 1:100 into fresh LB medium, grown to an OD_600_ of 0.6, and induced with 1 mM IPTG at 30 °C for 12 h. The cells were harvested by centrifugation and resuspended in 10 mL of wash buffer (50 mM HEPES-KOH pH 7.6, 7 mM beta-mercaptoethanol (BME), 1 M NH_4_Cl, 10 mM MgCl_2_, and 20 mM imidazole). After centrifugation, the pellets were suspended in lysis buffer (0.2 mg/mL lysozyme, 50 mM HEPES-KOH pH 7.6, 0.2% *v*/*v* Triton X-100, 1 M NH_4_Cl, 7 mM BME, 10 mM MgCl_2_, and 20 mM imidazole) and lysed by sonication. The soluble fraction was collected and filtered after centrifugation. The DOPARS was purified utilizing affinity chromatography following the manufacturer’s protocol using buffer A (50 mM HEPES-KOH pH 7.6, 7 mM BME, 10 mM MgCl_2_, 1 M NH_4_Cl) and buffer B (50 mM HEPES-KOH pH 7.6, 10 mM MgCl_2_, 100 mM KCl, 7 mM BME, 400 mM imidazole). The purified DOPARS was dialyzed using a 10 kDa molecular weight cutoff (MWCO) membrane for buffer exchange. After dialyzing into the stock buffer, the protein was stored at −80 °C (50 mM HEPES-KOH pH 7.6, 100 mM KCl, 30% glycerol, and 1 mM TCEP). The protein concentration was determined using the Pierce™ 660 nm Protein Assay kit (Thermo Fisher #22660, Waltham, MA, USA), and SDS-PAGE confirmed purity.

### 2.6. In Vitro ncAA Incorporation

To facilitate rapid and efficient protein synthesis, linear fragments generated by PCR were used as templates in the CFUPS system. A standard PCR reaction was conducted in a 50 μL volume comprising 1× Fastpfu buffer, 0.2 µM UF2 primers, 0.2 µM UR1 primers, 0.2 mM dNTPs, 2.5 U Fastpfu polymerase, and 1 ng plasmid. The PCR reaction was performed as follows: 2 min at 95 °C, 30 cycles of 20 s at 95 °C, 20 s at 55 °C, and 40 s at 72 °C, and then a 5 min extension at 72 °C. The band size was verified by agarose gel electrophoresis.

A CFUPS reaction with a final volume of 20 μL was performed at 30 °C for 3 h. The reaction mixture included a 35% cell extract based on the BL21(DE3) S-AAF3BF3 strain, 600 ng linear template, 1 × reaction buffer (40 mM HEPES-KOH, 130 mM potassium glutamate, 2 mM DTT, 2 mM 20 AA, 18 mM magnesium acetate, 1 mM putrescine, 1.5 mM spermidine, 0.34 mM NAD, 0.5 mM of each CTP, GTP, and UTP, 0.3 mM CoA, 170 µg/mL of tRNA (Sigma #10109541001, St. Louis, MI, USA), 34 µg/mL of folinic acid calcium, and 33.33 mM PEP), 5 µM DOPARS, and 1 mM DOPA. The synthesis was monitored at 30 °C for 3 h using a fluorescence spectrophotometer (excitation at 485 nm and emission at 535 nm).

To minimize the impact of the cell extract on subsequent enzyme activity, the proteins synthesized by CFUPS were purified. The 20 µL CFUPS reaction system was supplemented with 160 µL binding buffer (10 mM Tris-HCl pH 8.0, 1 mM EDTA, and 150 mM NaCl) and 20 µL of BeaverBeads™ Magrose Strep-Tactin (BEAVER #70808-250, Suzhou, China). The mixture was incubated at 4 °C for 30 min, followed by centrifugation to discard the supernatant. The pellet was washed three times with 200 μL of binding buffer. Subsequently, the pellets were incubated with 60 µL of elution buffer (2.5 mM biotin, 10 mM Tris-HCl pH 8.0, 1 mM EDTA, 150 mM NaCl, and 0.05% Tween-20) at 4 °C for an additional 30 min. The purified protein was then eluted and collected into a clean centrifuge tube for subsequent experiments. The purity of the protein was confirmed by WB.

### 2.7. sfGFP Quantitative Expression

To estimate the expression of the fluorescent protein in CFUPS, standard fluorescence curves were prepared according to the literature [17]. After the cell-free reaction, a 20 µL reaction sample was dispensed into a black opaque 384-well plate (Corning #3764, New York, NY, USA). The fluorescence was measured at 535 nm using a Spark multimode microplate reader (TECAN, Waltham, MA, USA) with an excitation wavelength of 485 nm. At least three independent experiments were conducted.

### 2.8. Mass Spectrometry Analysis

To obtain sufficient protein, a 200 µL CFUPS system was conducted at 30 °C for 3 h, and the protein was then purified based on the manufacturer’s protocol (BEAVER #70808-250, Suzhou, China). Subsequently, the protein was buffer-exchanged into 20 mM NH_4_HCO_3_ using an Amicon^®^ Ultra centrifugal filter (Millipore #UFC503096, Billerica, MA, USA). Then, the samples were sent to the College of Pharmacy Instrument Testing Center at Tianjin University for mass spectrometry analysis. The resulting spectra were deconvoluted using the Maximum Entropy deconvolution algorithm within the software.

### 2.9. Isothermal Titration Calorimetry (ITC)

DOPARS was dialyzed in buffer C (50 mM HEPES-KOH pH 7.6, 100 mM KCl, 1 mM TCEP). The concentration of the DOPARS was determined using the Pierce™ 660 nm Protein Assay kit (Thermo Fisher #22660). The binding experiment was performed in the MicroCal ITC200 system (Malvern, UK) at 25 °C. In brief, 80 µL of the ligand solution (either Tyr or DOPA at 800 µM) was loaded into the syringe and titrated into 280 µL of DOPARS protein (80 µM) in the sample cell. A total of 20 injections were carried out at the rate of 0.5 µL s^−1^ injection and 180 s intervals with a paddle stirring of 750 rpm. The data were collected using MicroCal Concat ITC software (version 1.0) and analyzed with MicroCal Origin software (version 7.0).

### 2.10. DOPA-Containing Protein Reverse Transcription Assay

To evaluate the impact of DOPA incorporation on reverse transcriptase (RT) activity, a two-step RT-PCR assay was conducted at varying temperatures according to a previous study [18]. In brief, the reverse transcription reaction was carried out in a 0.2 mL PCR tube with a final volume of 20 μL. The reaction mixture comprised 1× RT buffer (50 mM Tris-HCl pH 8.3, 75 mM KCl, 3 mM MgCl_2_, and 10 mM DTT), 0.125 mM dNTP, 0.1 µM MSR-R4 primer, and 2 µL the pull-down FrM4-DOPA. The reaction was performed as follows: 45 min at 37 °C or 55 °C, then 10 min at 85 °C. To quantify the cDNA synthesis yields, a standard PCR reaction was performed in a 20 μL volume containing 1 × PCR buffer, 0.2 mM dNTP, 0.2 µM Taq-F1 primer, 0.2 µM Taq-R1 primer, 0.1 U/µL Easytaq DNA polymerase (TransGen #AP111, Beijing, China), and 0.2 µM Probe-MS probe. The thermocycling conditions were as follows: an initial period of 5 min at 94 °C followed by 40 cycles of 30 s at 94 °C, 30 s at 55 °C, and 20 s at 72 °C. The fluorescence signals were measured during the extension phase.

The threshold cycle (Ct) at which the fluorescence significantly exceeds the background was determined by the QuantStudio Real-Time PCR software (version 1.2) (Applied Biosystems #Q6, Waltham, MA, USA). The impact of DOPA-containing FrM4 on activity was evaluated using the ΔCt method. The ΔCt value for each sample was calculated by subtracting the Ct value of the reference gene from the Ct value of the target gene. The experiments were independently repeated at least three times.

## 3. Results

### 3.1. Establishment of CFUPS Without Class I Release Factor

**Figure 1:**
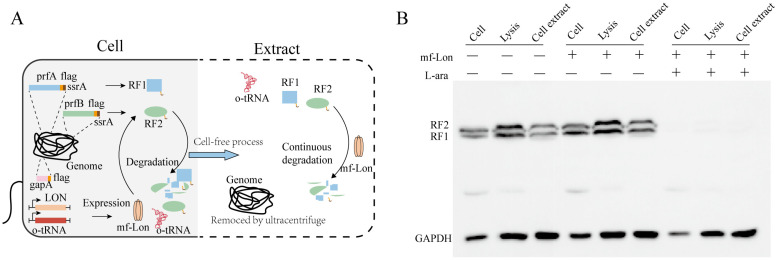
Establishment of CFUPS without Class I release factor. (**A**) The Class I release factor maintained a constant steady state within the cells due to the equilibrium between synthesis and degradation. After centrifuging, the genome is removed and endogenous gene expression is terminated. Throughout the cell extraction process, the Class I release factors were continuously degraded by the *mf*-Lon protein. (**B**) Western blotting analysis of the degradation of Class I release factors. During the preparation of the cell extract, the Class I release factors were analyzed via anti-Flag Western blotting, with GAPDH serving as the internal reference.

To establish a Class I release factor-free CFUPS system, we employed *mf*-Lon protein from the *Mesoplasma florum* for the specific degradation of pdt3-tagged proteins, as described previously [14]. The Class I release factor is an essential protein in bacteria, and its knock-out will significantly impact cell growth. CFUPS is a technology based on cell extract that facilitates in vitro protein synthesis without considering cellular activity or the influence of cell membranes. This system provides a robust platform for inducing *mf*-Lon expression in vivo and degrading the target protein in vivo and in vitro. To facilitate identification, the pdt3 tag was added to the ends of the *prfA* and *prfB* genes, with an additional flag tag inserted between pdt3 and the target gene. The *mf*-Lon was expressed in the BL21(DE3) S-AAF3BF3 strain with 1 mM L-arabinose. During the preparation of the cell extract, the genome was removed by centrifugation at 30,000× *g* after the cell lysis, halting Class I release factor transcription (Figure 1A). The results demonstrated that the Class I release factor was degraded in the presence of L-arabinose and *mf*-Lon (Figure 1B). These findings collectively establish that the CFUPS system was successfully established without the Class I release factor. Consequently, the cell extract will be utilized for further studies.

### 3.2. Optimized Incorporation of DOPA in CFUPS

**Figure 2:**
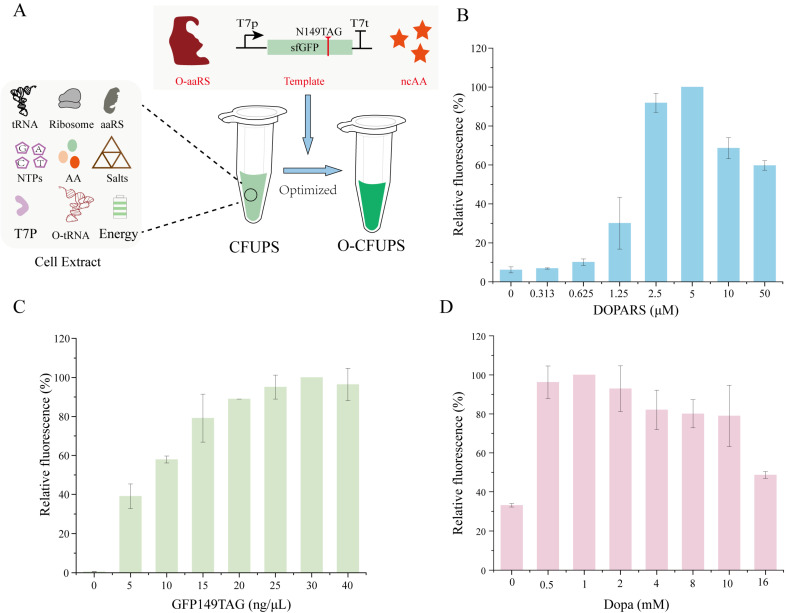
Optimized incorporation of DOPA in CFUPS. (**A**) Optimized incorporation of DOPA in CFUPS. The cell extract composition included tRNA, ribosomes, aminoacyl-tRNA synthetases (aaRSs), nucleotide triphosphates (NTPs), amino acids (AAs), salts, T7 RNA polymerase, energy components, and O-tRNA. The concentrations of O-aaRS, template, and ncAAs (highlighted in red) were specifically optimized within CFUPS. (**B**) Optimization of the DOPARS concentration. (**C**) Optimization of the template concentration. (**D**) Optimization of the ncAA concentration. The maximum fluorescence was calculated by identifying the highest fluorescence intensity within each experimental group, which was designated as 100%. The relative fluorescence was normalized relative to the maximum fluorescence.

Several aaRS/tRNA pairs for site-specific incorporation of DOPA into proteins using GCE have been reported [19,20,21,22]. However, most of these translational systems exhibit misincorporation issues in the DOPA assay medium (DAM) [16]. Recently, a highly orthogonal pair, *Methanocaldococcus jannaschii* tyrosyl-tRNA synthetase/tRNA_CUA_, was found to maximize DOPA incorporation in DAM-based assays. Therefore, the orthogonal system was combined with CFUPS without Class I release factors to implement DOPA incorporation of various stop codons. To enhance DOPA incorporation, several strategies need to be optimized based on CFUPS (Figure 2A). The concentration of aaRS directly influences the rate of protein translation, as aaRS is essential for amino acid activation and tRNA aminoacylation during translation. To maximize the synthesis efficiency of the target protein, the DOPARS tyrosyl-tRNA synthetase from *Methanocaldococcus jannaschii* was purified via affinity chromatography and added to CFUPS to promote DOPA incorporation (Supporting Figure S1). The concentration of DOPARS was then measured using the Pierce™ 660 nm Protein Assay kit based on a standard curve (Supplementary Figure S2). The superfolder green fluorescent protein (sfGFP) with an amber codon at position 149 was used as a model protein to monitor DOPA-containing protein synthesis by fluorescence detection. The yield increased with increasing DOPARS concentration, peaking at 5 μM, after which it gradually decreased. This trend is consistent with previous studies [23], indicating that continuous increases in aaRS concentration do not indefinitely boost protein production. In summary, the optimal DOPARS concentration was 5 μM.

To improve protein expression levels, the influence of template concentration on protein production was investigated. We aimed to boost protein expression by adjusting the template concentration in CFUPS reactions. The results demonstrated a saturation point when the template concentration was increased from 0 ng/μL to 40 ng/μL. The protein yield initially increased with more templates, stabilizing after reaching saturation. sfGFP expression reached its peak at a template concentration of 30 ng/μL (Figure 2C).

Next, the effect of DOPA concentration on protein expression was evaluated using an sfGFP mutant containing an amber codon at position 149 and fluorescence assays. The ncAA concentrations are known to significantly influence ncAA incorporation. To test the reaction, different concentrations of DOPA were added to CFUPS to measure the fluorescence level of sfGFP. The fluorescence value initially increased with increasing DOPA concentration, peaking at 1 mM, then decreasing (Figure 2D). Using the optimized system, DOPA incorporation into sfGFP resulted in a protein expression of 2.24 µg in a 20 μL reaction system based on the sfGFP standard curve (Supporting Figure S3A,B).

### 3.3. DOPA-Containing Protein Expression

**Figure 3:**
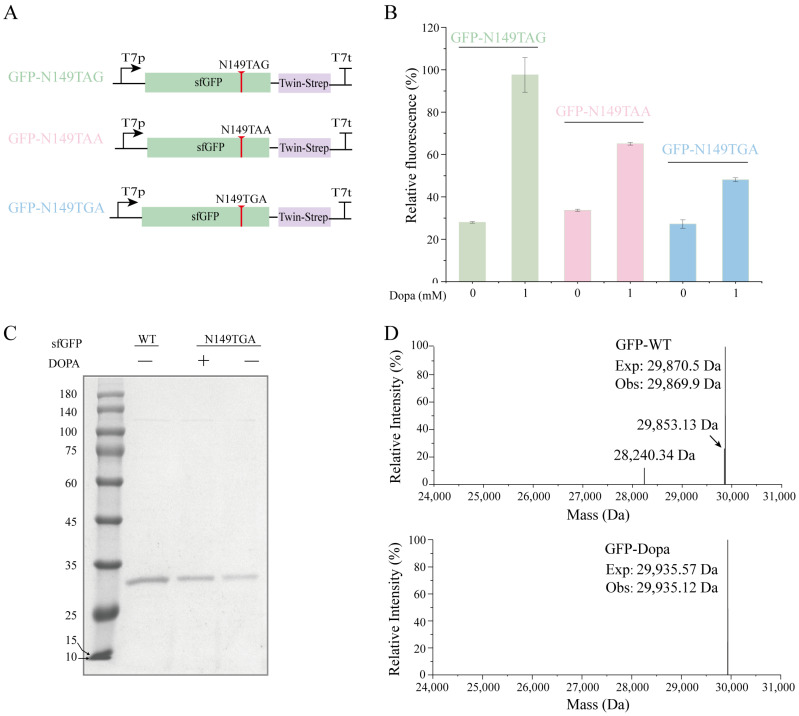
Incorporation efficiency and fidelity of DOPA. (**A**) Molecular characterization of different stop codons in sfGFP. (**B**) Incorporation efficiency and fidelity of DOPA in different stop codons. (**C**) SDS-PAGE analysis of WT-sfGFP and DOPA-containing sfGFP. (**D**) ESI-MS spectra of WT-sfGFP and sfGFP-149DOPA proteins. The percentage of fluorescence was normalized relative to the WT-sfGFP.

Using an optimized DOPA incorporation system, we investigated DOPA incorporation efficiency at three stop codons (TAG, TGA, and TAA) at the 149 positions of sfGFP (Figure 3A). Three *Mj*tRNAs (*Mj*tRNA_CUA_, *Mj*tRNA_UCA_, *Mj*tRNA_UUA_) are genetically modified to decode the stop codons TAG, TGA, and TAA, respectively. Cell extracts were prepared using overexpressed *Mj*tRNAs and the *mf*-Lon protein for DOPA incorporation. The results indicated that the incorporation efficiency of DOPA varied significantly across the different stop codons compared to wild-type sfGFP (WT-sfGFP). Notably, the incorporation efficiency of TAG stop codons was up to 97.6%; in contrast, the incorporation efficiency of TAA and TGA stop codons was less than 70%. Consequently, the DOPARS/*Mj*tRNA_CUA_ pair was employed for subsequent DOPA incorporations (Figure 3B). Furthermore, the incorporation of DOPA into multiple positions in the protein was also investigated. The results demonstrated that the fluorescence value of the protein decreased with the increase in the number of DOPA incorporations. Additionally, there was a homogeneous incorporation efficiency of only 39.6% when three DOPA molecules were incorporated (Supporting Figure S4). Importantly, fluorescence was still detectable in the absence of DOPA, suggesting potential non-specific incorporation of DOPA in CFUPS (Figure 3B). Meanwhile, the incorporation efficiency of DOPA was measured based on BL21(DE3) cell extract using the optimized system, with N49TAG as a template. The results showed that the incorporation efficiency in the presence and absence of DOPA was 0%, which was significantly lower than that observed in BL21(DE3) S-AAF3BF3 (Supporting Figure S5). To assess the accuracy of DOPA incorporation by the DOPARS/*Mj*tRNA_CUA_ pair, we purified sfGFP proteins using magnetic beads. The results demonstrated the presence of a single band with an approximate molecular mass of 29.8 kDa using SDS-PAGE analysis (Figure 3C). To further assess the accuracy of the DOPA incorporation, we conducted characterization through ESI-MS analysis. The results showed a single peak at 29,935 Da, consistent with the expected mass of sfGFP-N1149DOPA in the presence of DOPA (Figure 3D). Conversely, the dominant peak was observed at 29,918 Da in the absence of DOPA, which suggested the incorporation of Tyr at N149 when compared to the molecular weight of WT-sfGFP (29,870 Da) (Figure 3D, Supporting Figure S6). These findings establish the DOPARS/*Mj*tRNA_CUA_ pair as a reliable system for DOPA incorporation, demonstrating satisfactory amber suppression efficiency, high relative fidelity, and low absolute fidelity. Consequently, this suppression pair was used for further studies.

### 3.4. Measurement of the Affinity of DOPARS for DOPA

ITC is the gold standard for quantifying the release or absorption of heat while binding small molecules to biomolecules [24]. To elucidate the fidelity of the orthogonal system during DOPA incorporation, we determined the affinity of DOPARS for DOPA and Tyr using ITC (Figure S7A). The thermodynamic parameters and titration curve indicated that the affinity of DOPARS for DOPA was 11.7 μM (Figure S7B), consistent with the affinity of the corresponding substrates identified by aaRSs, as previously reported [25]. However, the titration curves indicated that DOPARS does not bind to the original amino acid counterpart of Tyr (Figure S7C). This may be attributed to mutations in four residues (L65E, H70A, D158A, and I159A) lining the amino acid binding pocket of the *Mj*TyrRS [16]. Additionally, the observed negative ΔH (−388.3 cal/mol) and positive ΔS (21.3 cal/mol/K) values suggest that the reaction is driven by both entropy and enthalpy changes, in line with previous research [26]. Surprisingly, the results showed negligible binding affinity of DOPARS for tyrosine, as observed in the ITC experiments. However, it appeared inconsistent with the misincorporation of tyrosine in the absence of DOPA (Figure 3C). DOPARS is not merely a substrate binder but an enzyme, and its catalytic activity must be characterized in the context of transition-state kinetics rather than equilibrium binding. The binding affinity under equilibrium conditions (as measured by ITC) may not fully reflect the enzyme’s ability to process tyrosine during the catalytic cycle.

### 3.5. Incorporation of L-DOPA into FrM4 RT in CFUPS

**Figure 4:**
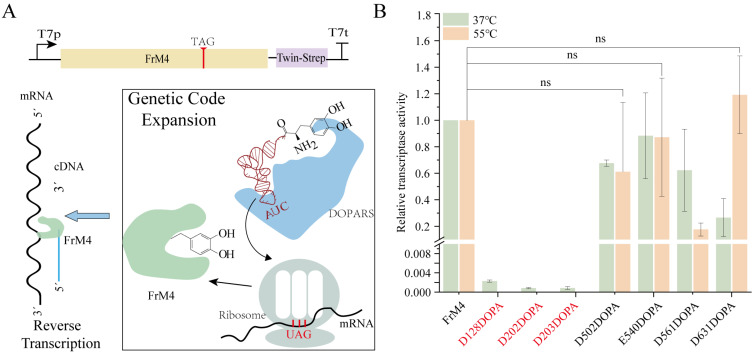
Incorporation of L-DOPA into FrM4. (**A**) DOPA incorporation into FrM4 using GCE based on CFUPS. (**B**) The effect of DOPA incorporation into the active site of FrM4 on its activity was observed at 37 °C and 55 °C. Reverse transcription yields of variants at different temperatures were determined by qPCR. Relative yields refer to the number of cDNA copies (assuming 100% PCR efficiency) produced by the FrM4. The red mark indicates that the reverse transcriptional efficiency of the mutation is not detected at 55 °C. The values are based on at least three independent experiments. (All error bars represent mean ± SD. ns: no statistical difference as determined by two-tailed *t*-tests).

After incorporating DOPA into the model protein sfGFP with high specificity and high relative fidelity using GCE, we explored its potential applications. DOPA has been demonstrated to bind metal ions [27]. It is hypothesized that the incorporation of DOPA into the metal binding site of FrM4 using GCE will generate better FrM4 variants. The residues D128, D202, and D203 have been identified as the active sites of the RNA-dependent polymerase, while residues D502, E540, D561, and D631 have been determined to be the active sites of RNase H. It is imperative to note that these sites all require binding to the metal ion Mg^2+^ to exhibit better catalytic function. To facilitate the purification of FrM4, we incorporated a Twin-strep tag at its C-terminus (Figure 4A). The FrM4 was purified using magnetic beads after the CFUPS reaction and was analyzed by Western blot. The results demonstrated no significant differences in the expression of all variants (Supporting Figure S8). To assess the activity of the FrM4-DOPA, we employed the two-step RT-qPCR method. The activities of the alternative variants were generally decreased compared to the parent, except for D631DOPA, which demonstrated similar activity to the parent at 55 °C (Figure 4B). These findings suggest that DOPA is unable to fully substitute for the function of native negatively charged residues (Asp/Glu) in metal coordination. A probable explanation for this is the size difference between DOPA and Asp/Glu, as well as the distinct chemical properties of the catechol moiety compared to the carboxylate group. Although the results did not meet the initial expectations, they highlight the challenges of engineering functional metalloenzymes using ncAAs and provide a foundation for future optimization efforts.

## 4. Discussion

The process of translation termination is typically dependent on three specific termination factors: RF1, RF2, and Release Factor 3 (RF3) in prokaryotic organisms. The codon preference of the Class I release factors, with RF1 recognizing only TAG and TAA, and RF2 recognizing TGA and TAA. The Class I release factors hydrolyze the ester bond between the peptide chain and tRNA, thereby facilitating the release of nascent peptide chains [28]. RF3 is a GTP-dependent enzyme that functions by assisting in the dissociation of RF1 and RF2 from ribosomes, which facilitates the completion of the termination process. However, it should be noted that in the absence of RF1 and RF2, translation termination can still be accomplished through several alternative mechanisms [29]. In the absence of RF1 and RF2, Alternative Ribosome Rescue Factor B (ArfB) and Peptidyl-tRNA Hydrolase (Pth) can synergistically facilitate translation termination. ArfB hydrolyzes peptidyl-tRNA through its intrinsic peptidyl-tRNA hydrolase activity, releasing the nascent peptide from the ribosome [30]. While Pth does not directly participate in translation termination, it hydrolyzes peptidyl-tRNA on the ribosome, aiding in nascent peptide release. Together, these complementary mechanisms ensure efficient ribosome and peptide release despite the absence of RF1 and RF2 [15]. ArfB and Pth do not exhibit codon-specific preferences, suggesting that they may interact with the ribosome in a more general manner, possibly by rescuing stalled ribosomes or facilitating peptidyl-tRNA hydrolysis independently of the stop codon identity. Meanwhile, the differing suppression efficiencies observed in the stop codons might be related to the specific tRNA modifications and the engineered anticodons [15]. This may explain the different inhibition efficiencies observed across the stop codons.

Stop codon suppression is a prevalent method for incorporating ncAAs into proteins. In this process, the incorporation of ncAAs directly competes with Class I release factors. To mitigate this competition, various strategies have been employed, such as altering TAG codons to TAA and knocking out the *prfA* gene [10], effectively reducing intracellular competition for ncAA incorporation. Additionally, some researchers have used affinity tags to selectively remove RF1 [31]. However, these methods are generally complex and not widely adopted. To enhance the incorporation efficacy of ncAAs in CFUPS, we employed the specific degradation properties of *mf*-Lon on pdt3-tagged proteins to construct a CFUPS system without the Class I release factor. To optimize resource utilization for engineered protein synthesis, we transformed the host strain with orthogonal tRNA (o-tRNA) and prepared cell extracts containing o-tRNA. Furthermore, we purified DOPARS in vitro and introduced it into CFUPS to control the precise synthesis of ncAA-containing proteins more effectively. Consequently, modifying the target protein sequence is sufficient to insert ncAAs into the protein at any desired location in CFUPS, thereby enabling the rapid synthesis of ncAA-containing proteins.

## 5. Conclusions

In this study, we employed a Class I release factor-free CFUPS containing o-tRNA based on the specific degradation effect of *mf*-Lon on ptd3-tagged proteins. By precisely optimizing the concentrations of DOPARS, template, and ncAAs in the system, we observed a significant increase in the expression of DOPA-containing proteins. The fidelity of the DOPA-containing protein was verified by ESI-MS, and the underlying mechanism was further elucidated by ITC. Finally, we successfully incorporated the DOPA-containing protein into the FrM4 protein.

## Data Availability

The data and materials in this study are available from the corresponding author upon reasonable request.

## Supplementary Materials

The following supporting information can be downloaded at: https://www.mdpi.com/article/10.3390/biom15030382/s1, Table S1: A list of the DNA oligonucleotides used, Table S2: Base sequence of the protein used in this study, Figure S1: SDS-PAGE analysis of the DOPARS, Figure S2: Standard curve of protein concentration based on BSA, Figure S3: Standard curve of sfGFP based on the fluorescence value, Figure S4: The incorporation of DOPA into multiple positions in the protein was also investigated, Figure S5: Incorporation efficiency of DOPA in different cell extracts, Figure S6: ESI-MS spectra of DOPA-containing sfGFP in the absence of DOPA, Figure S7: The affinity of DOPARS for DOPA and Tyr measured by ITC, Figure S8: Western blot analysis of the FrM4-DOPA protein, Figure S9: Full image of Figure 1B. Western blotting analysis of the degradation of Class I release factors, Figure S10: Full image of Figure 3C. SDS-PAGE analysis of the sfGFP.

## Abbreviations

The following abbreviations are used in this manuscript:

CFUPS	Cell-free unnatural protein synthesis
ncAAs	Noncanonical amino acids
GCE	Genetic code extension
ITC	Isothermal titration calorimetry
DOPARS	*Methanocaldococcus jannaschii* Tyrosyl-tRNA Synthetase
DOPA	3,4-Dihydroxy-L-phenylalanine
