# Supplementary material for: Efficient Incorporation of DOPA into Proteins Free from Competition with Endogenous Translation Termination Machinery
Source: Biomolecules. 2025 Mar 6;15(3):382. doi: 10.3390/biom15030382 (PMC11939889; doi:10.3390/biom15030382)
Supplement: Supplementary file 1 [file biomolecules-15-00382-s001.zip › biomolecules-3404905-supplementary.pdf]

Supporting Information

Efficient Incorporation of DOPA into Proteins free from Competition with Endogenous Translation Termination Machinery

Youhui Yang [1,2], Yingchen Wang [1,2], Zhaoguan Wang [1,2] and Hao Qi [1,2,*]

[1] School of Chemical Engineering and Technology, Tianjin University, Tianjin 300072, China
[2] Key Laboratory of Systems Bioengineering (Ministry of Education), Tianjin University, Tianjin 300072, China
* Correspondence: haoq@tju.edu.cn

Table S1. DNA oligonucleotides used are listed.

Oligo name	Sequence (5′-3′)
UR1	CCAATCCGGATATAGTTCCTCCTTTC
URB1	Biotin-CCAATCCGGATATAGTTCCTCCTTTC
UFB1	Biotin-ATAGGGCTAGCAATAATTTTGTTTAACTTTAAG
UF2	GATCTCGATCCCGCGGCGCTAATACGACTCACTATAGGGCTAGCAATAATTTTGTTTAAC
MSR-R4	CCTCTAGCACACGGGGTGCAAT
Taq-F1	GTCGCGGTAATTGGCGC
Taq-R1	GGCCACGTGTTTTGATCGA
Probe-MS	FAM-AGGCGCTCCGCTACCTTGCCCT-BHQ1
D128-F	ACACCGTTCTGtagCTGAAAGACGCTTTCTTCTGCCTGCG
D202-F	TGCAGTACGTTtagGACCTGCTGCTGGCTGCTACC
D203-F	AGTACGTTGACtagCTGCTGCTGGCTGCTACCTCTGAACTGG
D502-F	CCTGGTACACCtagGGTTCTTCTTTCCTGCAGGAAGGTCAGC
E540-F	CTCAGCGTGCTtagCTGATCGCTCTGACCCAGGCTCT
D561-F	ACGTTTACACCtagTCTCGTTACGCTTTCGCTACCGCTC
D631-F	ACCGTATGGCTtagCAGGCTGCTCGTGAAGTTGCTACC
D128-R	GCGTCTTTCAGctaCAGAACGGTGTACCACTGGTGAGACG
D202-R	AGCAGCAGGTCctaAACGTACTGCAGCAGGATCAGGTC
D203-R	GCCAGCAGCAGctaGTCAACGTACTGCAGCAGGATCAGG
D502-R	AAAGAAGAACCctaGGTGTACCAGGTGTGGTCAGCG
E540-R	AGAGCGATCAGctaAGCACGCTGAGCAGAGGTACCAGCC
D561-R	GCGTAACGAGActaGGTGTAAACGTTCAGTTTTTTACCTTCAGCC
D631-R	CGAGCAGCCTGctaAGCCATACGGTTACCACGAGCTTCAG
prfA-F	AGATGGCGCAGGATGAACTG
prfA-R	TCGAGGAAGGCGTTACGTTC
prfB-F	CGGTACGGTTAACGTGCTGAC
prfB-R	CCGCGTTTGTTTATCCGGAAG
ssrA-F	GGTATACTTACCTTTACACATTGGGG
ssrA-R	GTAATCACCGATGGAGAATTTTGGTGG
gapA-F	ATGACTATCAAAGTAGGTATCAACGG
gapA-R	TTATTTGGAGATGTGAGCGATCAGG

Table S2. The base sequence of the protein used in this study

Protein	Sequence
sfGFP	ATGCGTAAAGGCGAAGAGCTGTTCACTGGTGTCGTCCCTATTCTGGTGGAACTGGATG<br>GTGATGTCAACGGTCATAAGTTTTCCGTGCGTGGCGAGGGTGAAGGTGACGCAACTAA<br>TGGTAAACTGACGCTGAAGTTCATCTGTACTACTGGTAAACTGCCGGTACCTTGGCCG<br>ACTCTGGTAACGACGCTGACTTATGGTGTTCAGTGCTTTGCTCGTTATCCGGACCATAT<br>GAAGCAGCATGACTTCTTCAAGTCCGCCATGCCGGAAGGCTATGTGCAGGAACGCACG<br>ATTTCCTTTAAGGATGACGGCACGTACAAAACGCGTGCGGAAGTGAAATTTGAAGGCG<br>ATACCCTGGTAAACCGCATTGAGCTGAAAGGCATTGACTTTAAAGAAGACGGCAATAT<br>CCTGGGCCATAAGCTGGAATACAATTTTAACAGCCACAATGTTTACATCACCGCCGAT<br>AAACAAAAAAATGGCATTAAAGCGAATTTTAAAATTCGCCACAACGTGGAGGATGGC<br>AGCGTGCAGCTGGCTGATCACTACCAGCAAAACACTCCAATCGGTGATGGTCCTGTTC<br>TGCTGCCAGACAATCACTATCTGAGCACGCAAAGCGTTCTGTCTAAAGATCCGAACGA<br>GAAACGCGATCATATGGTTCTGCTGGAGTTCGTAACCGCAGCGGGCATCACGCATGGT<br>ATGGATGAACTGTACAAATAA
DOPARS	ATGGACGAGTTCGAGATGATCAAACGCAACACCAGCGAAATCATCAGCGAAGAAGAA<br>CTGCGCGAAGTGCTGAAAAAAGACGAGAAGAGCGCCTATATTGGCTTCGAACCGAGC<br>GGCAAAATTCATCTGGGCCACTACCTGCAGATCAAGAAGATGATCGATCTGCAGAACG<br>CGGGCTTCGATATCATCATTGAACTGGCGGATCTGGCGGCATACCTGAACCAGAAAGG<br>CGAACTGGACGAAATCCGCAAAATCGGCGACTACAACAAGAAAGTGTTCGAAGCGAT<br>GGGCTTGAAAGCGAAATACGTCTATGGCAGCGAATTTCAGCTGGACAAAGACTACAC<br>CCTGAACGTGTTTCGTCTGGCACTGAAAACCACCCTGAAACGTGCTCGTCGTTCTATGG<br>AACTGATTGCCCGCGAAGATGAAAACCCGAAAGTGGCGGAAGTGATCTATCCGATCA<br>TGCAGGTGAACGCCGCACACTATCTGGGCGTAGATGTAGCAGTTGGTGGCATGGAACA<br>GCGCAAAATCCACATGCTGGCTCGTGAACTGCTGCCGAAAAAAGTCGTGTGCATCCAC<br>AACCCGGTTTTAACGGGTCTGGATGGTGAAGGCAAAATGAGCAGCAGCAAAGGCAAC<br>TTTATCGCCGTGGATGATTCTCCGAAAGAAATCCGCGCGAAGATCAAAAAAGCGTATT<br>GCCCGGCAGGTGTAGTTGAAGGTAACCCGATCATGGAGATCGCGAAATACTTCCTGGA<br>ATACCCGCTGACCATTAAACGCCCGGAAAAATCCGGTGGTGATCTGACCGTGGACAGC<br>TACGAAGAACTGGAGAGCCTGTTCAAGAACAAAGAACCGCCGTTGAGGGCGCTGAAA<br>AATGCCGTTGCGGAAGAGCTGATCAAAATCCTGGAACCGATTCGCAAACGTCTGTAA
o-tRNAs	CCGGCGGTAGTTCAGCAGGGCAGAACGGCGGACTCTAAATCCGCATGGCAGGGGTTCAAATCCC<br>CTCCGCCGGACCA
FrM4	ATGACCTGGCTGTCTGACTTCCCGCAGGCTTGGGCTGAAACCGGTGGTATGGGTCTGGCTGTTCG<br>TCAGGCTCCGCTGATCATCCCGCTGAAAGCTACCTCTACCCCGGTTTCTATCAAACAGTACCCGA<br>TGTCTCAGGAAGCTCGTCTGGGTATCAAACCGCACATCCAGCGTCTGCTGGACCAGGGTATCCTG<br>GTTCCGTGCCAGTCTCCGTGGAACACCCCGCTGCTGCCGGTTAAAAAACCGGGTACCAACGACT<br>ACCGTCCGGTTCAGGACCTGCGTGAAGTTAACAAACGTGTTGAAGACATCCACCCGACCGTTCC<br>GAACCCGTACAACCTGCTGTCTGGTCTGCCGCCGTCTCACCAGTGGTACACCGTTCTGGACCTGA<br>AAGACGCTTTCTTCTGCCTGCGTCTGCACCCGACCTCTCAGCCGCTGTTCGCTTTCGAATGGCGTG<br>ACCCGGAAATGGGTATCTCTGGTCAGCTGACCTGGACCCGTCTGTCTCAGGGTTTCAAAAACTCT<br>CCGACCCTGTTCTGCGAAGCTCTGCACCGTGACCTGGCTGACTTCCGTATCCAGCACCCGGACCT<br>GATCCTGCTGCAGTACGTTGACGACCTGCTGCTGGCTGCTACCTCTGAACTGGACTGCCAGCAGG<br>GTACCCGTGCTCTGCTGCAGACCCTGGGTGACCTGGGTTACCGTGCTTCTGCTAAAAAAGCTCAG<br>ATCTGCCAGAAACAGGTTAAATACCTGGGTTACCTGCTGAAAGAAGGTCAGCGTTGGCTGACCG<br>AAGCTCGTAAAGAAACCGTTATGGGTCAGCCGACCCCGAAAACCCCGCGTCAGCTGCGTCGTTT<br>CCTGGGTCGTGCTGGTTTCTGCCGTCTGTGGATCCCGGGTTTCGCTGAAATGGCTGCTCCGCTGTA<br>CCCGCTGACCAAAACCGGTACCCTGTTCGAATGGGGTCCGGACCAGCAGAAAGCTTACCAGGAA<br>ATCAAACAGGCTCTGCTGACCGCTCCGGCTCTGGGTCTGCCGGACCTGACCAAACCGTTCGAACT<br>GTTCGTTGACGAAAAACAGGGTTACGCTAAAGGTGTTCTGACCCAGAAACTGGGTCCGTGGCGT<br>CGTCCGGTTGCTTACCTGTCTAAAAAACTGGACCCGGTTGCTGCTGGTTGGCCGCCGTGCCTGCG<br>TATGGTTGCTGCTATCGCTGTTCTGACCAAAGACGCTGGTAAACTGACCATGGGTCAGCCGCTGG<br>TTATCCTGGCTCCGCACGCTGTTGAAGCTCTGGTTAAACAGCCGCCGGACCGTTGGCTGTCTAAC<br>GCTCGTATGACCCACTACCAGGCTCTGCTGCTGGACACCGACCGTGTTCAGTTCGGTCCGATCGT<br>TGCTCTGAACCCGGCTACCCTGCTGCCGCTGCCGGAAGAAGGTCTGCAGCACGACTGCCTGGAC<br>ATCCTGGCTGAAGCTCACGGTACCCGTCCGGACCTGACCGACCAGCCGCTGCCGGACGCTGACC<br>ACACCTGGTACACCGACGGTTCTTCTTTCCTGCAGGAAGGTCAGCGTAAAGCTGGTGCTGCTGTT

ACCACCGAAACCGAAGTTGTTTGGGCTAAAGCTCTGCCGGCTGGTACCTCTGCTCAGCGTGCTGA
ACTGATCGCTCTGACCCAGGCTCTGAAAATGGCTGAAGGTAAAAAACTGAACGTTTACACCGAC
TCTCGTTACGCTTTCGCTACCGCTCACATCCACGGTGAAATCTACCGTCGTCGTGGTTGGCTGACC
TCTGAAGGTAAAGAAATCAAAAACAAAGACGAAATCCTGGCTCTGCTGAAAGCTCTGTTCCTGC
CGAAACGTCTGTCTATCATCCACTGCCCGGGTCACCAGAAAGGTAACCGTGCTGAAGCTCGTGGT
AACCGTATGGCTGACCAGGCTGCTCGTGAAGTTGCTACCCGTGAAACCCCGGAAACCTCTACCCT
GCTGTAA

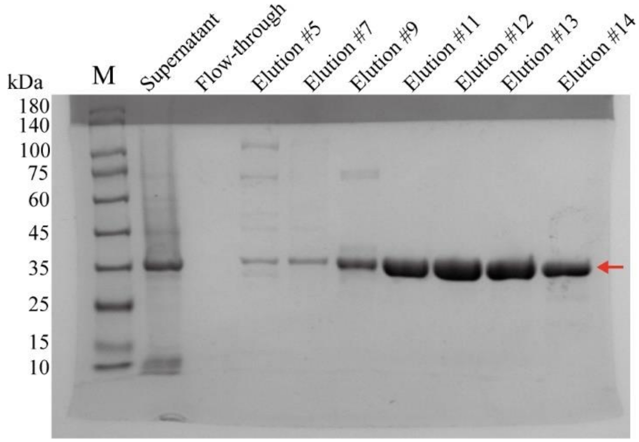

Fig. S1 **SDS-PAGE analysis of the DOPARS.**

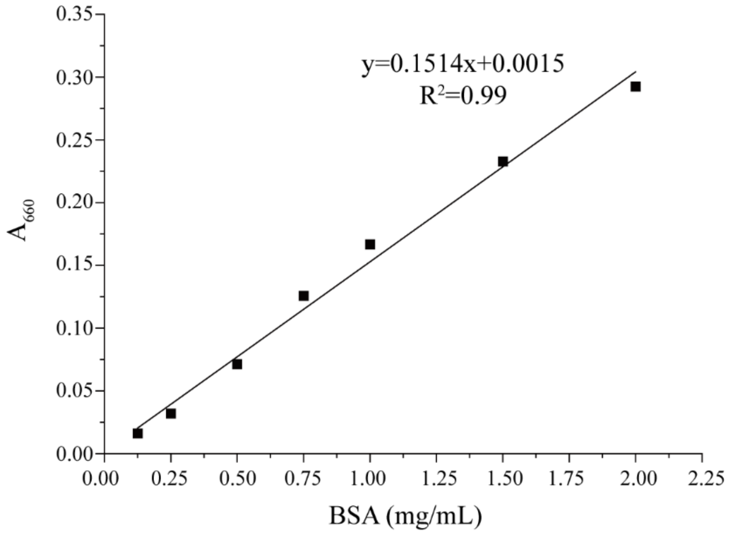

Fig. S2 The standard curve of protein concentration based on BSA.

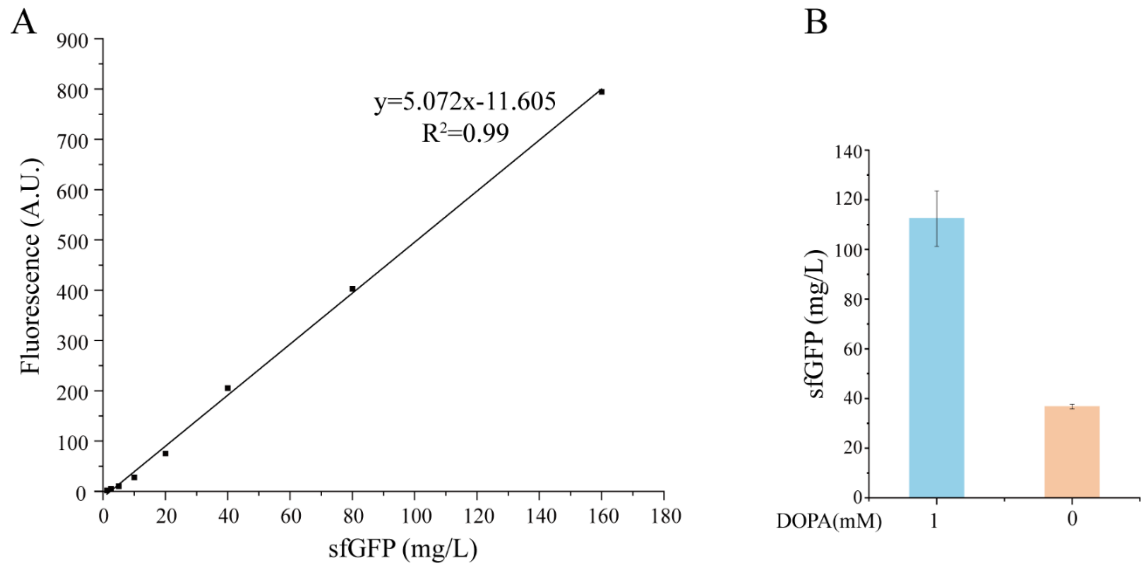

Fig. S3 The standard curve of sfGFP based on the fluorescence value.

(A) The standard curve of sfGFP. (B) sfGFP-149DOPA protein expression

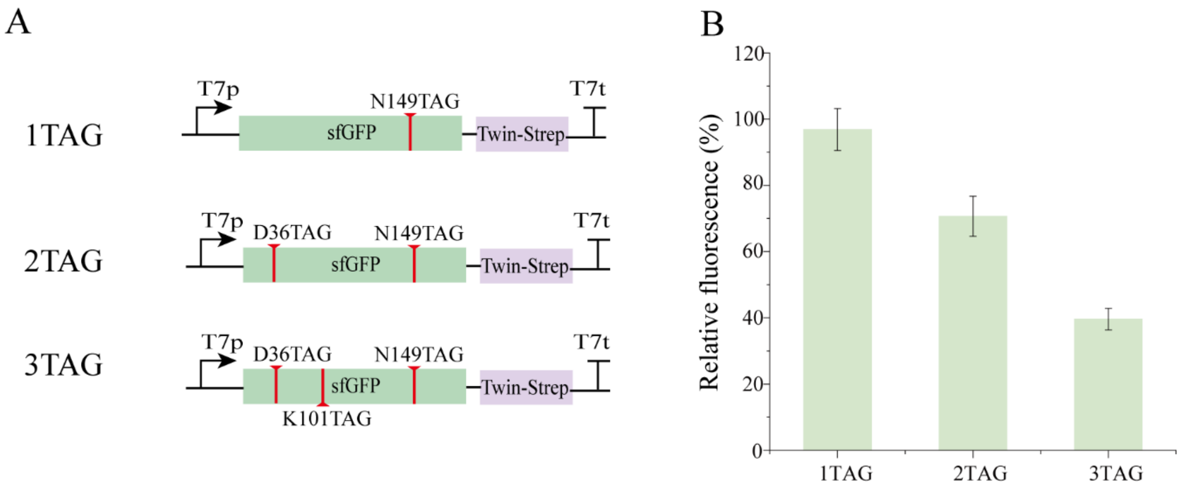

Fig. S4 The incorporation of multiple positions with DOPA into the protein was also investigated. (A) Protein expression in molecular characterization. (B) The relative fluorescence activity of DOPA at multiple sites in the protein. The percentage of fluorescence was normalized relative to the WT-sfGFP.

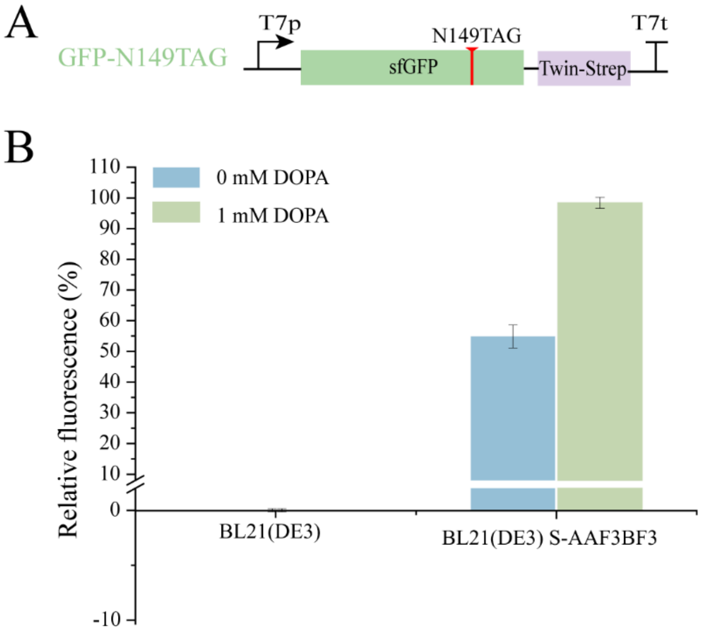

Fig. S5 The incorporation efficiency of DOPA in different cell extracts

(A) The template of DOPA incorporation. (B) The incorporation efficiency of DOPA in different cell extracts. (B) Incorporation efficiency and fidelity of DOPA in different stop codons. The percentage of fluorescence was normalized relative to the WT-sfGFP.

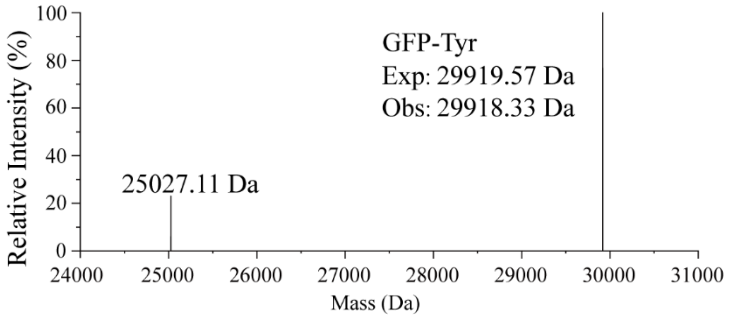

Fig. S6 ESI-MS spectra of DOPA-containing sfGFP in the absence of DOPA.

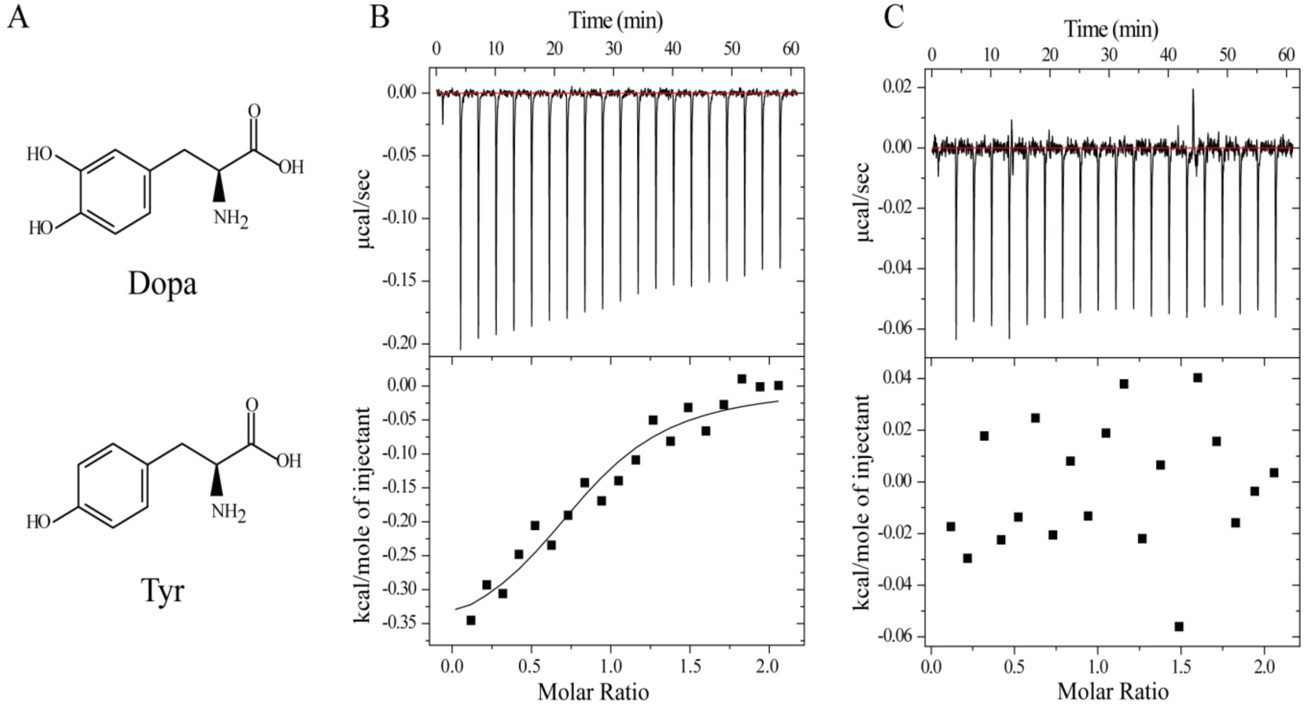

Fig. S7 The affinity of DOPARS to DOPA and Tyr was measured by ITC. (A) The molecular formula of DOPA and Tyr. (B) ITC measured the thermodynamic parameters of DOPARS and DOPA. (C) ITC measured the thermodynamic parameters of DOPARS and Tyr.

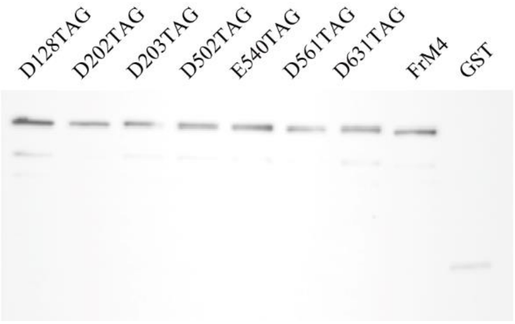

Fig. S8 Western Blot analysis of the FrM4-DOPA protein.

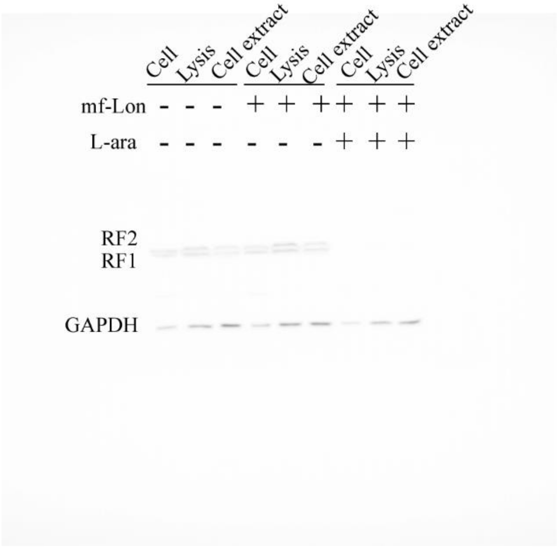

Fig. S9 Full image of Fig. 1B. Western blotting analysis of the degradation of class-I release factors.

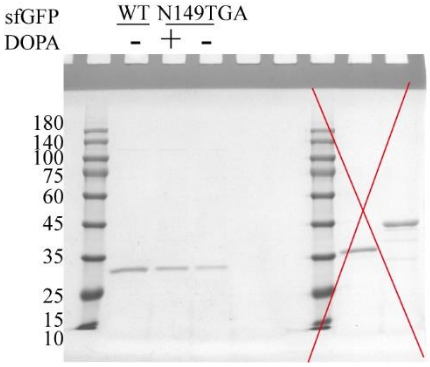

Fig. S10. Full image of Fig. 3C. SDS-PAGE analysis of the sfGFP.
