# Supplementary figures and images for: Efficient Incorporation of DOPA into Proteins Free from Competition with Endogenous Translation Termination Machinery
Source: Biomolecules. 2025 Mar 6;15(3):382. doi: 10.3390/biom15030382 (PMC11939889; doi:10.3390/biom15030382)

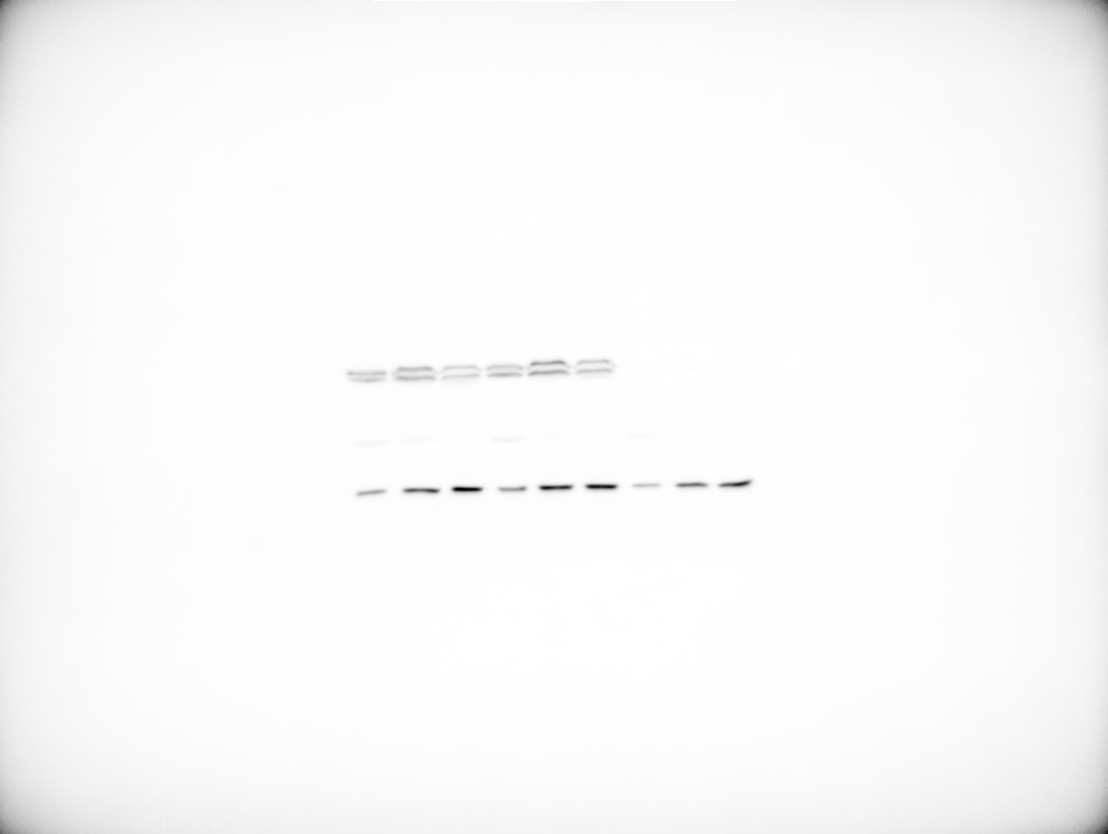

Supplement: Supplementary file 1 [file biomolecules-15-00382-s001.zip › biomolecules-3404905-original-images/Figure 1B-Full image.tif]

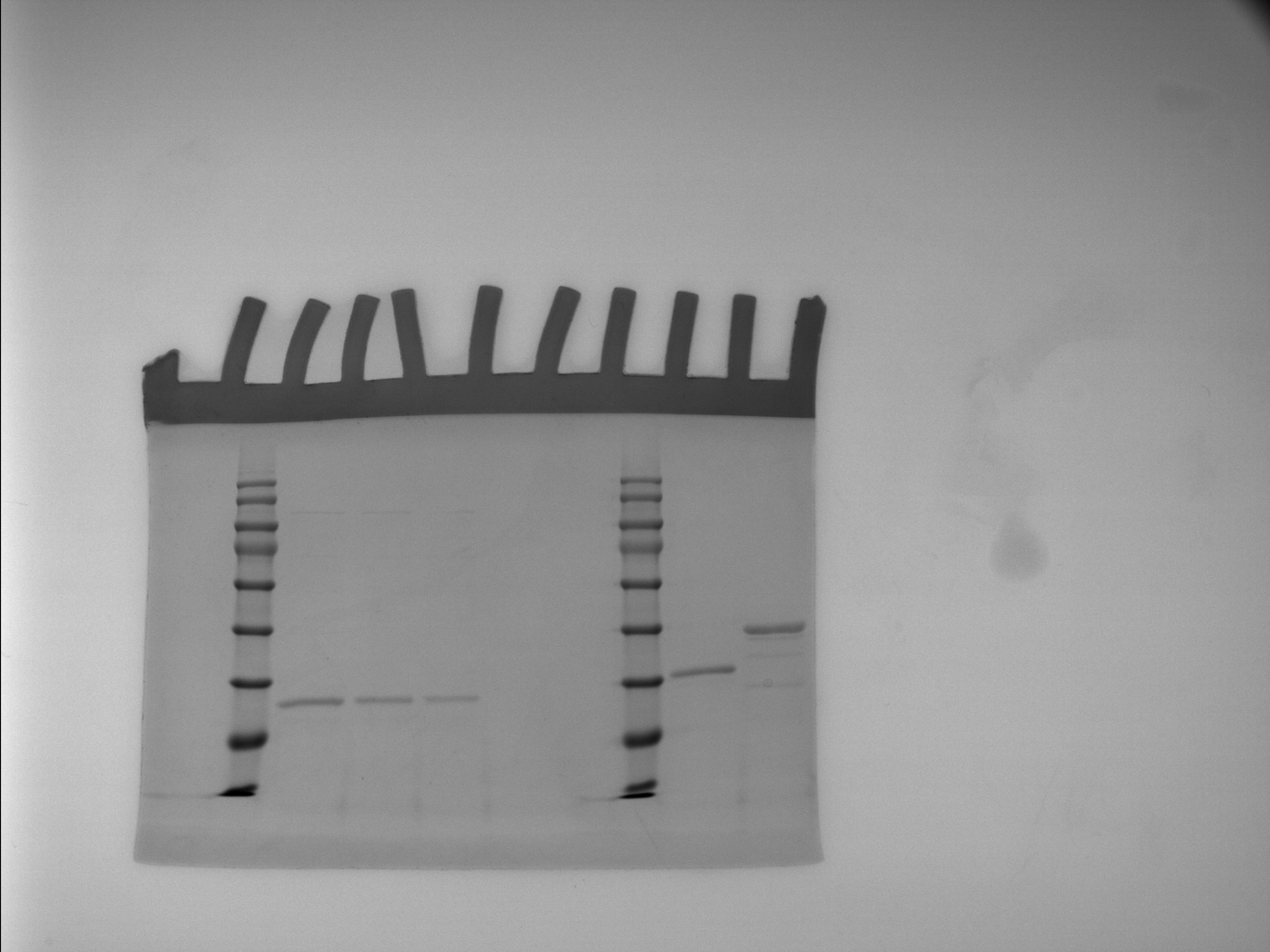

Supplement: Supplementary file 1 [file biomolecules-15-00382-s001.zip › biomolecules-3404905-original-images/Figure 3C-Full image.tif]

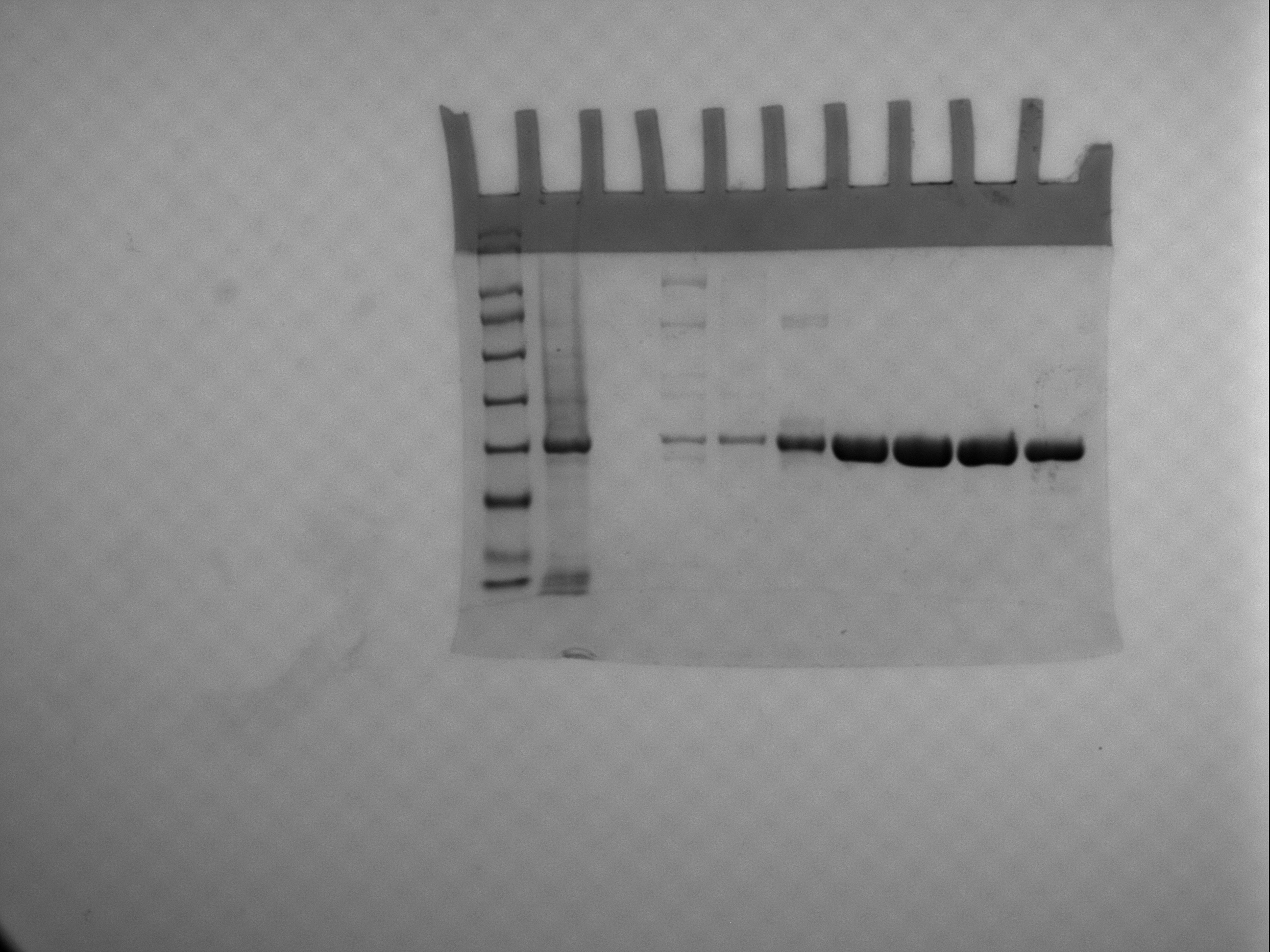

Supplement: Supplementary file 1 [file biomolecules-15-00382-s001.zip › biomolecules-3404905-original-images/Figure S1-Full image.tif]

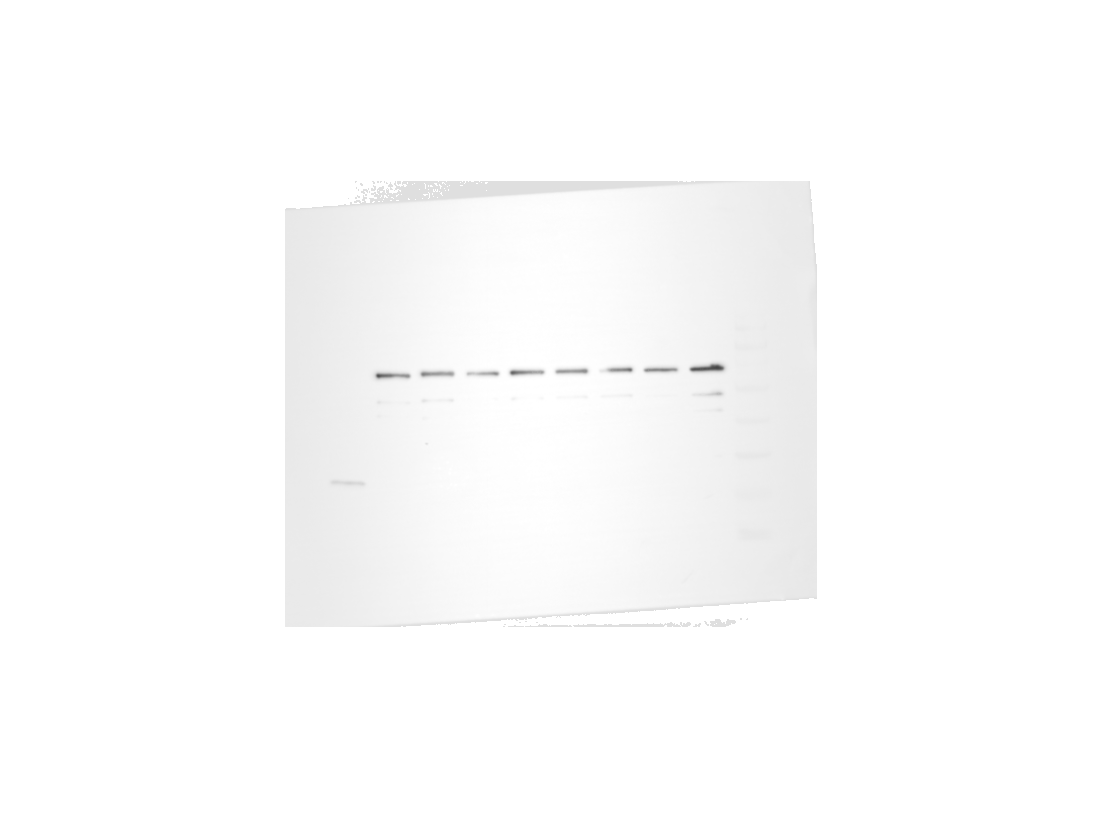

Supplement: Supplementary file 1 [file biomolecules-15-00382-s001.zip › biomolecules-3404905-original-images/Figure S6-Full image.tif]
